# Supplementary material for: Comparing miRNA structure of mirtrons and non-mirtrons
Source: BMC Genomics. 2018 Feb 9;19(Suppl 3):114. doi: 10.1186/s12864-018-4473-8 (PMC5836839; doi:10.1186/s12864-018-4473-8)
Supplement: Supplementary file 1 — The particular examples of the 3′ end shifting inside miRNA. (DOCX 16 kb) [file 12864_2018_4473_MOESM1_ESM.docx]

The examples of the 3′ end shifting inside miRNA in related pre-miRNAs: *tgu-let-7b-5p* and *aca-let-7b-5p*; *tgu-let-7b-3p* and *mml-let-7b-3p*. The same shifts can be observed by comparing nucleotide contexts in figures 2A and 2C from the paper of Starega-Roslan et al. [1].

>tgu-let-7b (MI0013703)

ucuaa aU ucaggguagugauuu

5' cagg GAGGUAGUAGGUUGUGUGGUU u

|||| |||||||||||||||||||||

3' gucC UUCCGUCAUCCAACAUAUCaa g

---ua -C uagaggacuaacccc

>aca-let-7b (MI0018702)

uagcucugg u ucaggguagucauuu

5' caagg GAGGUAGUAGGUUGUGUGGUu u

||||| |||||||||||||||||||||

3' guuCC UUCCGUCAUCCAACAUAUCaa g

--------a - uagaggacuaacccc

>mml-let-7b MI0007573

U ---- ---a u

5' cgggg GAGGUAGUAGGUUGUGUGGUU uc gggcag g

||||| ||||||||||||||||||||| || |||||| a

3' guCCC UUCCGUCAUCCAACAUAUCaa ag cccguu u

- uaga acuc g

1. Starega-Roslan J, Witkos TM, Galka-Marciniak P, Krzyzosiak WJ. Sequence features of Drosha and Dicer cleavage sites affect the complexity of isomiRs. *International journal of molecular sciences*. 2015;16:8110-8127.
