# Supplementary material for: Comparing miRNA structure of mirtrons and non-mirtrons
Source: BMC Genomics. 2018 Feb 9;19(Suppl 3):114. doi: 10.1186/s12864-018-4473-8 (PMC5836839; doi:10.1186/s12864-018-4473-8)
Supplement: Supplementary file 2 — Change of the pre-miRNA secondary structure reduces a pair of long overhangs to nearly canonical one. (DOCX 15 kb) [file 12864_2018_4473_MOESM2_ESM.docx]

The example how the secondary structure model can be changed to reduce a pair of long overhangs to nearly canonical one: the upper model is the secondary structure of the pre-miRNA mmu-mir-467c (MI0005512) from miRBase [1], the bottom model is proposed here.

> mmu-mir-467c (MI0005512):

- - g ----AA CA -- auuuua

5' cc uuu ugcaU GUGCGUG UGUAUA UGUGuguau u

|| ||| ||||| ||||||| |||||| ||||||||| g

3' gg aag gcgua cacguac aCAUAU ACACACAUA c

c u a cagaca ac CC CAUAUA

Proposed structure:

--- c A C C ua

5' gug aUA GUG GUG AUGUAUAUGUGug u

||| ||| ||| ||| ||||||||||||| a

caC UAU CAC CAC UACAUAUAcguau u

3' aca A C A A uu

1. Griffiths-Jones S. The microRNA Registry. Nucleic Acids Research. 2004;32(Database issue):D109–D111. http://doi.org/10.1093/nar/gkh023
