# Supplementary material for: Comparing miRNA structure of mirtrons and non-mirtrons
Source: BMC Genomics. 2018 Feb 9;19(Suppl 3):114. doi: 10.1186/s12864-018-4473-8 (PMC5836839; doi:10.1186/s12864-018-4473-8)
Supplement: Supplementary file 3 — The model of independent overhangs and ist results. (DOCX 24 kb) [file 12864_2018_4473_MOESM3_ESM.docx]

**Table S1. The guanine frequencies in the neighborhood of 5' end of 3' miRNAs for different pairs of overhang lengths.** In each cell of the table the second number corresponds to the start position of the miRNA, the first and the third numbers – to its surrounding positions inside and outside miRNA, respectively. The guanine content is minimal at the beginning miRNA position. The amount of G in the surrounding positions is not sufficient to explain the excess of the 1nt/1nt and 3nt/3nt overhang pairs (table S3).

|  |  | Drosha | | |
| --- | --- | --- | --- | --- |
|  | Overhang length, nt | 1 | 2 | 3 |
| Dicer | 1 | 0.173  0.103 0.297 | 0.158  0.098  0.303 | 0.241  0.063  0.329 |
|  | 2 | 0.229  0.068  0.274 | 0.203  0.081  0.300 | 0.196  0.061  0.303 |
|  | 3 | 0.259  0.071  0.212 | 0.199  0.108  0.327 | 0.124  0.067  0.381 |

**Table S2. The matrix of overhang relative frequencies for the model of independent overhangs.** Let α (β) be a relative frequency of observing the overhang length 1nt shorter (longer) than the canonical 2nt length. Then the matrix of the two-parametric model of overhang lengths is the following:

|  |  | Drosha | | |
| --- | --- | --- | --- | --- |
|  | Overhang length, nt | 1 | 2 | 3 |
| Dicer | 1 | α^2^ | α | αβ |
|  | 2 | α | 1 | β |
|  | 3 | αβ | β | β^2^ |

The mean of the Dicer (X) and Drosha (Y) overhang lengths can be calculated as following: $M\left( X \right)=M\left( Y \right)=\sum_{i} x_{i}p_{x_{i}}=\frac{1}{\gamma}\left( 1\times\left( \alpha^{2}+\alpha+\alpha\beta\right)+2\times\left( \alpha+1+\beta\right)+3\times\left( \alpha\beta+\beta+\beta^{2} \right) \right)=\frac{1}{\gamma}\left( \alpha^{2}+3\alpha+4\alpha\beta+5\beta+2+3\beta^{2} \right)$, where $\gamma=\alpha^{2}+2\alpha+2\alpha\beta+2\beta+\beta^{2}+1$.

The mean of the joint lengths of the Dicer and Drosha overhangs (XY) can be calculated as following: $M\left( XY \right)=\sum_{i} \sum_{j} x_{i}y_{j}p_{ij}=\frac{1}{\gamma}\left( \alpha^{2}+2\alpha+3\alpha\beta+2\alpha+4+6\beta+3\alpha\beta+6\beta+9\beta^{2} \right)=\frac{1}{\gamma}\left( \alpha^{2}+4\alpha+6\alpha\beta+12\beta+4+9\beta^{2} \right)$, where $\gamma=\alpha^{2}+2\alpha+2\alpha\beta+2\beta+\beta^{2}+1$.

By direct calculation one can see that $M\left( XY \right)=M\left( X \right)M\left( Y \right)$ and therefore the overhang lengths are independent within this model.

|  |  | Drosha | | |
| --- | --- | --- | --- | --- |
|  | Overhang length, nt | 1 | 2 | 3 |
| Dicer | 1 | 0.132 (0.065) | 0.257 (0.255) | 0.035 (0.036) |
|  | 2 | 0.254 (0.255) | 1 (1) | 0.153 (0.142) |
|  | 3 | 0.037 (0.036) | 0.131 (0.142) | 0.046 (0.020) |

**Table S3. The matrix of the observed overhang lengths for animal.** The numbers of observations are normalized to the number of duplexes with canonical overhangs, i.e. with both 2nt overhangs (2271). The expected values according to the model of independent overhang lengths (table S2) are given in brackets with α = 0.255 (shortening), β = 0.142 (elongation). The α and β values are obtained by ordinary least squares without taking into account the cells 1,1 and 3,3 (fitting all the cells produces the similar result). One can see that the expected frequencies match the observed frequencies except the pairs 1,1 and 3,3 which occur twice as often as predicted.
