# Supplementary material for: Comparing miRNA structure of mirtrons and non-mirtrons
Source: BMC Genomics. 2018 Feb 9;19(Suppl 3):114. doi: 10.1186/s12864-018-4473-8 (PMC5836839; doi:10.1186/s12864-018-4473-8)

To show up that our results are robust against the overhang definition we additionally analyzed two subsets.

First subset contains 3743 miRNA duplexes in which the 5′ end of the 5′ miRNA is paired (figure 1, table 1), in this case the overhang lengths are unambiguously. For example:

> mmu-mir-27b

AUUG ugau

5' aggugcAGAGCUUAGCUG GUGAACag uggu

|||||||||||||||||| |||||||| ||| u

3' uccaCGUCUUGAAUCGGU CACUUguu gccu

--GA --uc

Second subset contains 1969 miRNA duplexes in which the 5′ end of the 5′ miRNA is unpaired but its second nucleotide is not (figure 2). For example:

>hsa-mir-34a

- -a -- - ug uU - A -guga a

5' ggcc gc ugug ag uuucu GGCAGUGU CUU GCUGGUUGUu gc a

|||| || |||| || ||||| |||||||| ||| |||||||||| ||

3' ccgg ug gcac uc gaaga CCGUCAUA GAA CGACUAACga ug u

c gg uu g gu UC U - aggaa a

**Table 1. The matrix of the observed lengths of unambiguously overhangs.** The observation numbers are normalized to the number of duplexes with both canonical 2nt overhangs (1396). The expected values according to the model of independent overhang lengths are given in brackets with α = 0.234 (shortening) and β = 0.140 (elongation). The α and β values are fitted by ordinary least squares without taking into account the cells 1,1 and 3,3. Note that these α and β coefficients are quite close to their values for the full dataset.

|  |  | Drosha | | |
| --- | --- | --- | --- | --- |
|  | Overhang length, nt | 1 | 2 | 3 |
| Dicer | 1 | 0.108 (0.055) | 0.251 (0.234) | 0.033 (0.033) |
|  | 2 | 0.217 (0.234) | 1 (1) | 0.144 (0.140) |
|  | 3 | 0.037 (0.033) | 0.135 (0.140) | 0.049 (0.020) |

**Fig 1. The length distributions of the 3′ hanging end of the 3′ miRNA for Subset 1 (only 0-5nt hanging ends and 1-3nt Dicer overhangs are shown).** For the short hanging ends (less than canonical 2nt) 1nt Dicer overhangs are observed more often than 3nt ones while for the long hanging ends they go by opposites. The mirtron number is too small to make statistical conclusions.


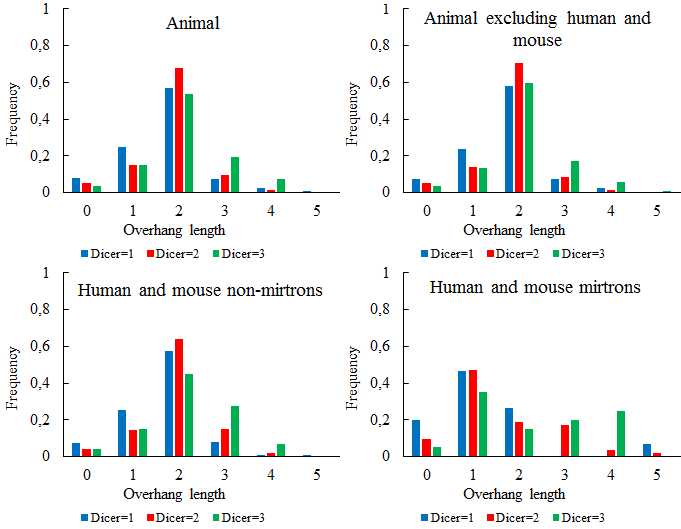


**Fig 2. The length distributions of the 3′ hanging end of the 3′ miRNA for Subset 2 (only 0-5nt hanging ends and 1-3nt Dicer overhangs are shown).** The results reproduce the tendency of overhang lengths on Fig 1; note that the canonical overhang corresponds to the 3nt 3′ hanging end. The mirtron number is too small to make statistical conclusions.


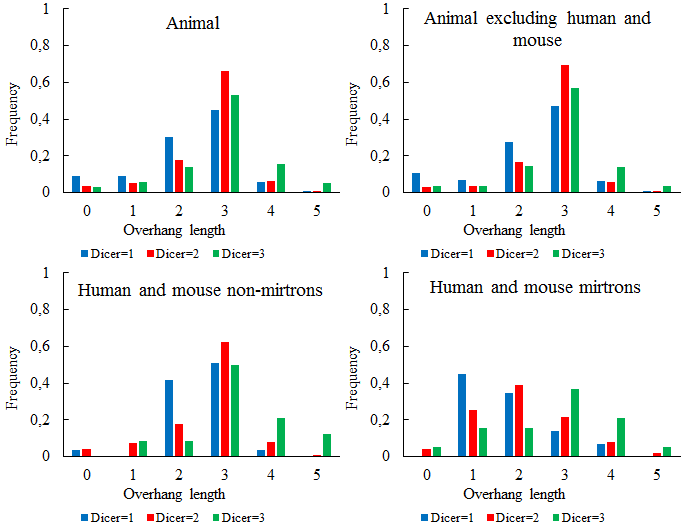

Supplement: Supplementary file 4 — Overhang length dependence are robust against the overhang definition. (DOCX 62 kb) [file 12864_2018_4473_MOESM4_ESM.docx]
