# Supplementary figures and images for: Comparing miRNA structure of mirtrons and non-mirtrons
Source: BMC Genomics. 2018 Feb 9;19(Suppl 3):114. doi: 10.1186/s12864-018-4473-8 (PMC5836839; doi:10.1186/s12864-018-4473-8)

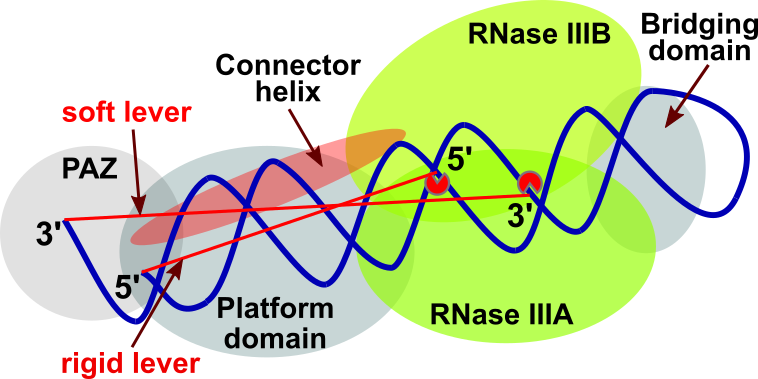

Supplement: Supplementary file 5 — The scheme of the proposed 2-lever model for Dicer. (PNG 91 kb) [file 12864_2018_4473_MOESM5_ESM.png]
