# Supplementary material for: Comparing miRNA structure of mirtrons and non-mirtrons
Source: BMC Genomics. 2018 Feb 9;19(Suppl 3):114. doi: 10.1186/s12864-018-4473-8 (PMC5836839; doi:10.1186/s12864-018-4473-8)
Supplement: Supplementary file 6 — The branchpoint locations across mirtrons and introns. (DOCX 31 kb) [file 12864_2018_4473_MOESM6_ESM.docx]

**Table S1. The fractions of branchpoint site sequences that fit U2 basepairing models (see [1] for details).**

| model | Human and mouse mirtrons, % | Human and mouse introns, % |
| --- | --- | --- |
| canonical | 9,33 | 10,15 |
| canonical2nt | 6,67 | 6,40 |
| canonicalC | 4,00 | 3,83 |
| TRAYTRY | 4,00 | 6,36 |
| TRANYTRY | 13,33 | 7,11 |
| TRANNYTRY | 5,33 | 10,40 |
| none | 51,33 | 42,39 |
| circle | 1,33 | 4,41 |
| template_switching | 4,67 | 8,95 |

**Figure S1. Branchpoint position relative to the 3′ splice site for the human and mouse mirtrons and introns.** Position «-50» contains all observations with distances ≥ 50nt. 70.4% (84.6%) of intron (mirtron) branchpoints fall into the expected region (10-40 nucleotides upstream from the 3′ splice site).


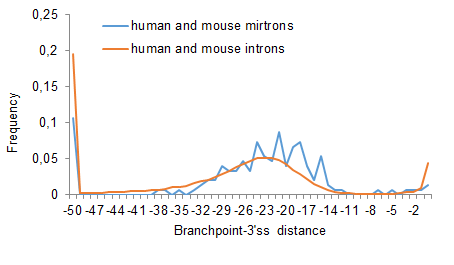


1. Taggart AJ, Lin CL, Shrestha B, Heintzelman C, Kim S, Fairbrother WG. Large-scale analysis of branchpoint usage across species and cell lines. *Genome research*. 2017;27:639-649.
