# Supplementary material for: Comparing miRNA structure of mirtrons and non-mirtrons
Source: BMC Genomics. 2018 Feb 9;19(Suppl 3):114. doi: 10.1186/s12864-018-4473-8 (PMC5836839; doi:10.1186/s12864-018-4473-8)
Supplement: Supplementary file 7 — The results of the stability test using MiRGeneDB database. (DOCX 95 kb) [file 12864_2018_4473_MOESM7_ESM.docx]

Below we present the results for the miRBase miRNAs whose identifiers are simultaneously presented in [1]. The annotated miRNA sequences in miRBase and in MirGeneDB may slightly differ. For convenience, here the pictures order is the same as in the main text of the article. The mirtrons data are not presented because there are only seven mirtrons in MirGeneDB.


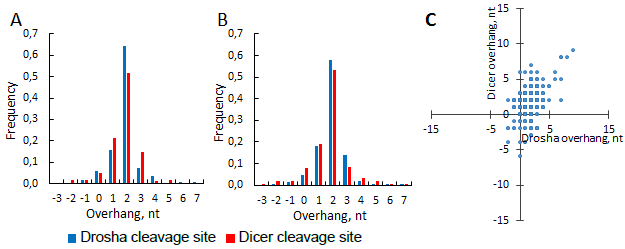


**Figure 1. The overhang lengths of miRNA duplexes.** The frequency of the overhang lengths of miRNA duplexes: animal miRNAs without human/mouse ones (A), human and mouse non-mirtrons (B). The overhang lengths occurrence of both cleavage sites for miRNA duplexes (C). Negative values correspond to an atypical 5' overhangs. The long overhangs are not shown on the panels A and B. Note that the overhang pairs 1,1 and 3,3 are still overrepresented.


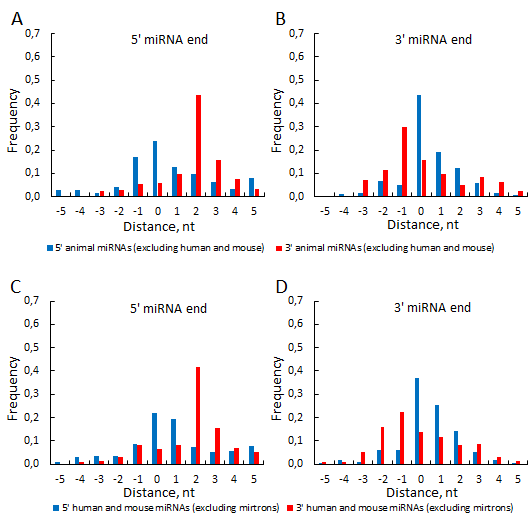


**Figure 2. Distance between miRNA end and its nearest single-stranded nucleotide in the same miRNA strand**. Considered are only those miRNA ends in which their terminal nucleotide is double-stranded. The frequencies of the 5′ miRNA ends are shown on panels A and C. The frequencies of the 3′ miRNA ends are shown on panels B and D. The data are presented for 5′ and 3′ miRNA sequences separately: for animal miRNAs excluding human and mouse ones (A and B) and for the human and mouse non-mirtrons (C and D). The positive values correspond to the distances to the nearest single-stranded nucleotide outside the miRNA. The negative values are the numbers of nucleotides that must be cut off from the miRNA to reach the nearest loop in the miRNA. The distance 0 is observed for those miRNA ends that are exactly at the boundary of the single-stranded region.


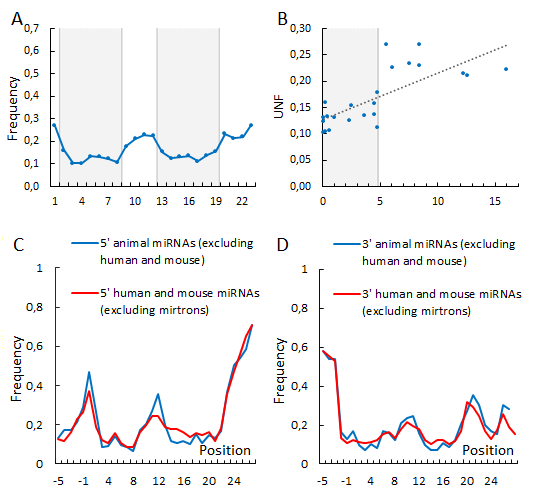


**Figure 3. The unpaired nucleotide frequency (UNF) across the miRNA sequence.** 5′ end of the miRNA starts from position one. Negative positions correspond to the miRNA flank. The UNF is not shown at the very ends of several long miRNAs. (A) Animal miRNAs. (B) The UNF dependence on the relative rate of nucleotide substitutions in animal miRNAs. The seed points concentrate near the very UNF-axis. Spearman’s rank correlation test was used to estimate the significance of the correlation between the UNF and the rate of nucleotide substitutions (ρ=0.79, P=8.16×10^-6^). (C-D) The UNF profile of 5′ miRNAs and of 3′ miRNAs of human and mouse non-mirtrons and of animal excluding human and mouse.

**
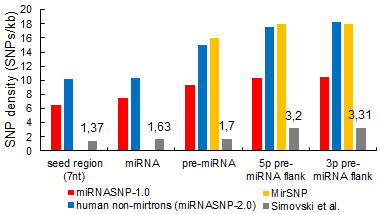
**

**Figure 4. SNPs in pre-miRNA**. SNP density in human pre-miRNAs and their flanking regions. Blue bars correspond to human non-mirtrons. The results are based on miRNASNP-2.0 (miRBase-19.0 and dbSNP137) [2]. Red bars were calculated by [3] using miRBase-16.0 and dbSNP132 (miRNASNP-1.0 database). Yellow bars were calculated by [4] using miRBase-18.0 and dbSNP135 (MirSNP database). Gray bars were calculated using the data of Simovski et al (<https://hyperbrowser.uio.no/hb/>) [5]. SNP density is shown separately for seed region, miRNA (without seed region), pre-miRNA (without miRNA) and upstream and downstream 1kb pre-miRNA flanks. The MirSNP data (yellow bars) provide the densities of the entire pre-miRNA sequence and of both 200bp pre-miRNA flanks. Gray bars fit to the region classification of Simovski et al., which differs from this paper. Gray pre-miRNA bar was calculated for the terminal loop. Gray pre-miRNA flanks were calculated for the 30bp pre-miRNA flanks starting from the miRNA borders.

References.

1. Fromm B, Billipp T, Peck L, Johansen M, Tarver JE, King BL, Peterson KJ. A uniform system for the annotation of vertebrate microRNA genes and the evolution of the human microRNAome. Annual review of genetics. 2015;49:213-242.
2. Gong J, Liu C, Liu W, Wu Y, Ma Z, Chen H, Guo Y. [An update of miRNA SNP database for better SNP selection by GWAS data, miRNA expression and online tools](http://www.ncbi.nlm.nih.gov/pubmed/?term=25877638). Database (Oxford). 2015; doi: [10.1093/database/bav029](https://dx.doi.org/10.1093%2Fdatabase%2Fbav029).
3. Gong J, Tong Y, Zhang HM, Wang K, Hu T, Shan G, Guo AY. Genome-wide identification of SNPs in microRNA genes and the SNP effects on microRNA target binding and biogenesis. Human mutation. 2012;33:254-263.
4. Liu C, Zhang F, Li T, Lu M, Wang L, Yue W, Zhang D. MirSNP, a database of polymorphisms altering miRNA target sites, identifies miRNA-related SNPs in GWAS SNPs and eQTLs. BMC genomics. 2012;13:1.
5. Simovski, 2017. Simovski B, Vodák D, Gundersen S, Domanska D, Azab A, Holden L, Johansen M. GSuite HyperBrowser: integrative analysis of dataset collections across the genome and epigenome. GigaScience, 2017;gix032.
